# Making Champs and Super-Champs—Current Views, Contradictions, and Future Directions

**DOI:** 10.3389/fpsyg.2017.00823

**Published:** 2017-05-26

**Authors:** David J. Collins, Aine Macnamara

**Affiliations:** Institute of Coaching and Performance, University of Central LancashirePreston, United Kingdom

**Keywords:** talent, development, mental skills, lifeskills, performance pathway

## Abstract

In our 2016 paper (Collins et al., 2016a), we proposed that superchamps (athletes who have achieved the highest level in their sport) were differentiated from their less successful counterparts by their use of positive proactive coping and a “learn from it” approach to challenge. This skill-based focus to talent development (TD) is supported extensively in the literature (e.g., MacNamara et al., 2010a,b) and suggests that the differences between levels of adult achievement relate more to what performers *bring to* the challenges than *what* they experience (Collins et al., 2016a). In this focused review we present and discuss a number of key concepts related to this paper and other parallel research in TD. We begin by presenting our pragmatic objectives and the importance of considering how we evaluate the research with an emphasis on its application to the applied setting. We then consider commonalities and differences in recent psychological approaches to TD, namely the experiential, attitudinal, and skill-based. The paper then provides further exploration of the Psychological Characteristics of Developing Excellence and their role in TD processes. We conclude with a consideration of future research and the application of research in TD. Reflecting our pragmatic stance as researchers, we hope that this focused review provides suggestions for progress and stimulates critical debate amongst practitioners, researchers and policy makers.

## Introduction—what are we trying to achieve?

From an elite sport perspective, talent development (hereafter TD) is an important process. Optimizing the pathway to produce the best possible senior athletes is, arguably, the lifeblood, of sports, and teams. The real-world experiences of young athletes follow a non-linear and dynamic trajectory and there is growing recognition that facing and overcoming a degree of challenge is desirable for aspiring elites and as such, should be recognized and employed. Indeed, in the 2016 paper “Superchampions, Champions and Almosts: Commonalities and differences on the rocky road” that is the focus of this review, superchamps (cf. Collins et al., 2016a; athletes who had achieved the highest level in their sport) were differentiated from their less successful counterparts by their use of positive proactive coping and a “learn from it” approach to challenge. This skill-based focus to TD is supported extensively in the literature (e.g., Gould et al., 2002; MacNamara et al., 2010a,b) and it would seem that the differences between eventual levels of adult achievement relate more to what performers *bring to* the challenges than *what* they experience. Interestingly, the mental side works as both outcome (leading to mentally strong competitors) and process (developing performers who can more fully exploit and benefit from the opportunities and inputs provided—Collins et al., 2016a).

As we detail later in the paper, TD is a popular topic and there have been numerous studies which have explored the optimum design and conduct of TD Environments or TDEs. Our focus is predominantly psychological, although we work hard to ensure a broad interdisciplinary perspective and to consider other domains. This importance notwithstanding, the present paper is focused on psychological constructs and there is certainly no shortage of investigations and papers in the domain. Of course, almost all of these studies (including our own) must be viewed as works in progress until prospective and longitudinal studies can be completed. The old adage that “correlation does not prove causation” is appropriate here. Furthermore, the extent to which any one factor could be uniquely causative, or predictive of something as complex and dynamic as talent development must also be questioned. Therefore, even robust longitudinal or experimental data are unlikely to support a single method solution.

Importantly, however, our work is pragmatic in its philosophy (cf. Giacobbi et al., 2005) and is aimed at making a difference. As “pracademics” we use the most suitable academic approaches to find solutions to real-world problems, whilst acknowledging the difficulty of finding truly causative factors in real-life settings. For the moment therefore, we offer the suggested guidelines with a note of caution. We return to this perspective in Section How it works indirectly. For the moment, we would stress that the pursuit of our pragmatic aim leads us to adopt the best answers available, on the basis of appropriate research; that is, work that suggests how differences can be made.

Accordingly, in this invited review (for which, due thanks) we consider how our Psychological Characteristics of Developing Excellence (PCDE) research and other related work informs the “making of a superchamp” against the backdrop of parallel strands in this important area, highlighting gaps and next steps needed. If we are critical of others' work, it is to explain why we have taken the line we have, and also to contextualize the professional judgement and decision making processes which we would suggest for practitioners. In other words, what ideas should they consider using and why.

## Where are we now?

### Current psychological approaches to TD. *Are* we all saying the same thing?

In a recent paper (Collins et al., 2016b), we suggested that **current psychological approaches to TD** could be classified into three groups namely, experience, attitudes, and skills. The first, experiential emphasis includes the recent UK Sport sponsored Great British Medallists study (Hardy et al., 2013) together with publications that arose from this report (e.g., Rees et al., 2013, 2016) and the resilience focused work of Sarkar et al. (e.g., Fletcher and Sarkar, 2012; Sarkar and Fletcher, 2014; Howells and Fletcher, 2015, 2016). The second covers ideas such as the Growth Mindset (Dweck, 2006) and the grit approach of Duckworth et al. (e.g., Duckworth et al., 2007, 2010; Eskreis-Winkler et al., 2014). The third approach, where our work is situated, sees skill development and deployment as *the* key element (e.g., Toering et al., 2009, 2011; MacNamara et al., 2010a,b; MacNamara and Collins, 2012; Honer and Feichtinger, 2016).

KEY CONCEPT 1 **Current psychological approaches to TD**

Current approaches to TD could be classified into three groups; namely, experience—what happened to the individual, attitudes—ideas such as growth mindset and grit, and skills—Psychological Characteristics of Developing Excellence development and deployment as *the* key element of development. We suggest that *all* are based on the development of skills but however the skills got there, it is *possession* and *confidence in possession* that makes the athlete.

We will critically consider the shortcomings of these approaches in subsequent sections. For the moment, however, we wish to focus on what links rather than differentiates these ideas; namely, the mechanisms through which any or all may convey advantage. In short, we apply a pragmatic philosophy as the main criterion. In fact, in terms of mechanism, we suggest that *all* are based on the development of skills in the individuals concerned, together with the confidence in personal ability to deploy them for positive effect. Put simply, however the skills got there, it is the *possession* and *confidence in possession* that makes the athlete (MacNamara and Collins, 2010). For practitioners, acceptance of this concept offers a clear way forwards. Certainly, this idea drives our emphasis on skills and confidence; simply put, if skills and confidence *are* the crucial causative factor, why would one leave this to chance, as with the experience approach, or hope that they grow from a specific attitude? Perhaps, this is what needs emphasizing by all concerned, at least if they are really focused on accentuating the benefit for performers, otherwise referred to as psychology *for* performance (cf. Collins and Kamin, 2012).

In fairness, we think that some of these authors acknowledge this. For example, as Sarkar et al. highlight in their paper “…adversity-related experiences offer potential developmental opportunities *if they are carefully and purposely harnessed*” (2015, p. 475. Our emphasis added). Accordingly, understanding how best to exploit challenging traumas seems to us a far more productive focus for applied researchers than the descriptive approach which is critiqued in the next section.

### And IF we are not saying the same thing, what needs to be considered in evaluating the evidence?

As stated in the Introduction, we have taken a number of epistemological and methodological decisions in our work, designed to develop, test, and refine concepts which help us to best achieve our stated purposes. These objectives are unashamedly pragmatic in nature so, as a consequence, the applied *implications* of research are an important part of our judgement criteria. Reflecting this, we would firstly consider what elements of the research listed above, all presented as part of an applied subject, are really equipped to make a justified contribution to TD practice. Clearly related issues, such as the target sample, methods used, etc., will also play a part in **evaluating the evidence**.

KEY CONCEPT 2 **Evaluating the evidence**

The applied capability of research is an important part of our judgement criteria. Unfortunately, the retrospective, individualistic and single methodologies that characterize much of the TD research limit the applicability of the advice offered. We propose the importance of a pragmatic approach to research in this area and the need for the triangulation of multiple measures in the field.

In terms of the *implications* criterion, and associated methodological and epistemological decisions, we would have to say that the picture is mixed. The grit related work of Duckworth et al. seems very positive in this regard, at least as far as it goes. A careful and progressive series of investigations offers a picture of a valid construct, with broad implications across populations and environments. The use of multiple measures, including real life outcomes (e.g., military academy performance; university success) with genuine meaning to participants are other strengths. The main challenge to grit in the current literature (Credé et al., 2016) seems to be with the construct itself; in part, that the positive effects of grit may be largely attributable to perseverance, a distinct but perhaps a subset construct of what is seen as grit *per se*. In sum, or at least for our present purpose, confusion seems apparent over what actually makes the effects happen. Some of these strengths and weaknesses are also apparent in the work of Dweck et al. on growth mindset. Once again, a full dissection of this work is not our purpose. For the moment, we would highlight the lack of clarity on *how* the mindset is developed and deployed to improve performance. Dweck's work is pleasingly clear on the consequences of having the correct mindset, but much less so on how this might be generated through a series of educational steps. We would like to extend this “construct clarity” criticism to the resilience approach as well. In this case there is an even more substantial literature base around the construct and its impact and although some authors (e.g., Sarkar and Fletcher, 2014) acknowledge the role of psychological skills in developing resilience, there is still little proposed about *how* this should happen in a TD setting. In all three cases, however, are we talking about a target outcome behavior rather than focusing on the process which will achieve it? It is worth noting that some of the authors cited above (confusingly in our opinion) see “their” construct as both outcome *and* process, while others do not even address this important distinction. Finally, to what extent do the constructs proposed offer a potential solution to the breadth of challenges which TDEs involve. So, for example, will grit or growth mindset or resilience alone provide a comprehensive “toolkit”? From another perspective, what do the purely descriptive accounts offered by the “life experience” perspective offer to coaches or other stakeholders in TD? In short, based on their findings, what should (indeed could) a coach do differently?

A second criterion would be to examine the clarity of information offered by TD authors on exactly how the construct is generated. Once again, the resilience work of Sarkar et al. must be questioned. A large part of their argument seems insufficiently critical of the role of trauma on mental resilience which, although implicit within their TD and sport literature, seems unsupported in mainstream psychology. Indeed, the literature seems clear that “post traumatic growth” will often benefit from intervention (Joseph et al., 2012), whilst recent work by Infurna and Luthar (2016) suggests that a resilient reaction to major life stressors is far less common than once thought. In sum, we would suggest that growth from challenge seems much more dependent on skills than the experience itself. A more parsimonious explanation seems to stress the importance of pre-existing skills to cope with the trauma or, at the very least, reflection skills and a facilitating reflector to learn the lessons *post hoc* (see the latest report from the National Scientific Council on the Developing Child, 2015). Our suggestion would be that, whilst successfully overcoming a life challenge can contribute to confidence, one had better go in with the skills at least partially present.

Our final point would be to question the use of “single” methodologies in many of the studies cited. We are very aware of this as a potential shortcoming, and have already stressed the need for triangulation of multiple measures in this field (Collins et al., 2016a,b). We (and others: see Males, 2016) are, therefore, increasingly concerned to see athlete autobiographies as the sole data source in studies of elite athletes. The approach has received some support in sport sociology (e.g., Stewart et al., 2011) but even they highlight some characteristics of this source which, for the TD pathway purposes we are discussing here, would seem disadvantageous. They report that “autobiographies, rather than seeking historical accuracy or objective truth, seek to offer deep insights into subjective expressions of experience…autobiographies emphasize not facts, but personal experiences and personal lives as cultural constructions” (p. 583). Furthermore, the retrospective nature of these data means that the status of the athlete will influence their perception of the route to the top. “Almosts” are likely to regard certain developmental challenges as traumatic while the same event might be recounted as non-traumatic by superchamps. The athlete's eventual success will undoubtedly color their perception of the pathway and this impression management might be even more of a factor for athletes still involved in the sport. We would highlight, however, that this retrospective “rosy glow” would seem more logical as minimizing perceptions of early challenges. In short, this is a complex situation which is unlikely to be well addressed by reading a filtered account of one's own memories.

At the very least, the highly *individual* perspective described would seem questionable as the basis for advising coaches on how to work *generally* with athletes. Even more recent work, suggesting that the post traumatic positive effects grow with time is once again based on a single measure; in this case, retrospective interview (Howells and Fletcher, 2016). So, given these issues, what characteristics should we look for in evaluating current, or designing future studies?

## How does the skills approach operate?

### How it works directly

As explained in our original paper (Collins et al., 2016a), we suggest that developing skills in an appropriately challenging environment appears to be a big element of the processes which can lead an athlete to Super-champ status. There is clearly room for debate on exactly which skills may most beneficially be taught, although we would suggest care in discriminating between contributory skills (such as the PCDEs) and outcomes such as mental toughness or resilience. Our particular blend has evolved over 20 years, with the current iteration presented in the left side of Table 1. This list has evolved from the original ideas of Terry Orlick (e.g., Orlick and Partington, 1988), and been refined as a result of several studies focused on talent development (e.g., MacNamara et al., 2010a,b). Parallel strands have examined characteristics of effective TDEs (e.g., Martindale et al., 2010) and the design and deployment of quantitative instruments to operationalise and track the constructs, such as the Psychological Characteristics of Developing Excellence Questionnaire or PCDEQ (MacNamara and Collins, 2011, 2012). In short, PCDEs are the product of a significant body of work, rather than a single investigation.

**Table 1:** **PCDEs and factors from the PCDEQ2**.

**PCDE Skills**	**PCDEQ2 Factors**
■ Commitment ■ Focus and distraction control ■ Realistic performance evaluation ■ Self-awareness ■ Coping with pressure ■ Planning and self-organization ■ Goal setting ■ Quality practice ■ Effective imagery ■ Actively seeking social support	1. Adverse response to failure (Fear of failure) 2. Imagery and active preparation 3. Self-directed control and management 4. Perfectionistic tendencies 5. Seeking and using social support 6. Active coping 7. Clinical indicators (e.g., eating disorders, anxiety, depression)

More recently, work has focused on the development of a second version of the questionnaire, the PCDEQ2, which measures these skills (Hill et al., in review), generating the important factors presented on the right of Table 1. In simple terms, the *skills* can be thought of as equipping the performer with a “hand of cards,” which are then deployed to achieve or counter the *factors*. Notably, four of these factors are positive (numbers 2, 3, 5, and 6) so athletes are encouraged to maximize them. Two are negative (1 and 7) so skills are deployed to counter and avoid whilst one (number 4) is “OK in moderation” or when expressed in certain ways.

As such, the main aims of the skills development approach are to build the skill set, develop the performer's ability to deploy subsets of skills to address challenges, and to ensure a confidence and commitment to doing this under pressure. As can be seen in Table 1, there is a good deal of overlap between the skills, a factor which we have come to accept as inevitable. What continuously emerges from our application of this approach, however, is that recognizing and exploiting these inter-skill similarities is a big part of the total skill set needed on the pathway. As a further and we suggest, essential advantage, the developing athlete is equipped with skills which can be used in different combinations to realize different aims. For example, not all challenges encountered will require resilience. Under certain circumstances, TD athletes must refuse to tolerate certain situations and seek alternatives, seeking and using input from others. Thus, debilitating performance anxiety might be addressed by use of imagery, focus and coping. As another example, athletes must learn to tolerate situations as “beyond their control” (cf. emotion-focused coping). So, tolerating a difficult coach or team mate might require the athlete to employ goal setting, social support, and self-awareness. The point is that adaptability necessitates a range of skills, useable to address a variety of challenges.

Based on these aims, and supported by our academic work (e.g., Collins et al., 2010), the developmental approach taken to skill development is key. Our experience of starting the develop–challenge-refine-retest process (what we colloquially refer to as TEACH-TEST-TWEAK-REPEAT—Collins et al., 2016b) has so far gone as young as 6 years old. We would suggest that starting early offers more chance to develop and embed skills, provides skills to counter roadblocks/life events before they occur and offers plenty of opportunity for gradually incremental progression. As the performer progresses both the complexity of challenge (and therefore the skills required) and the balance of challenge to support increases. This “periodization” of challenge is an important factor in the coach's planning. Of course, we are also very aware of the need to build skills proactively, in anticipation of the inevitably unforeseen challenges which will also occur. As such, it is important to consider the other factors, outside of the control of the individual athlete, that influence development and the importance of proactively preparing the athlete for these eventualities. In this case, depending on the nature of the challenge and its impact, coaches will often work in tandem with psychologists but they should almost always retain some role in the process to ensure transfer and relevance. Parental support and involvement is another important feature, facilitated by an early introduction of the principles, together with ongoing briefings and workshops. We have been using this approach in academy settings for a few years and look forward to soon testing the impact of this work in genuinely longitudinal studies which can consider both successes and failures against the progression of PCDE skills or lack thereof.

Finally, both the nature and the source of challenge must be noted. In this regard, it is interesting to note the way in which the balance of support and challenge is often presented by high status coaches as a crucial part of their armory. The challenge is often carefully considered but ruthlessly applied by the manager who takes the leading role (Times, 2016); an element which some authors have described as dark leadership (Cruickshank and Collins, 2015). Cleverly, however, support functions are often concurrently filled by other members of the support team, working under the manager/head coach's direction (Cruickshank and Collins, 2012; Cruickshank et al., 2013). Being able to use this approach with performers is clearly reliant on their possession of a robustness and self-driven adaptability, a characteristic which we see as the final target of the skills based approach of development.

### How it works indirectly

It is important to repeat that the PCDE list, although our current “best option,” is not the only consideration in how the skills-based approach affects developing athletes. For a start, other lists of skills could be employed, so long as they can be used in different combinations to address varied challenges rather than a more limited subset, such as persistence *or* attitude to growth *or* resilience. A related factor is that, through the use of the teach-test-tweak cycle, developing athletes get used to handling progressively harder challenges, accepting temporary setbacks whilst building a longer term approach. The resulting increase in self-esteem is a crucial contributor to the impact of the skills-based approach factor. Indeed, self-esteem has been shown as essential to conferring resilience to emotional distress (Johnson et al., 2017). As another key “value-added,” the approach fits well with the social aspects shown to characterize effective TDEs (e.g., Martindale et al., 2005; Henriksen, 2010) whilst also developing elements of high performance culture in the young athletes and support staff through the encouragement of a can-do approach.

As another parallel strand, these same mental factors have been seen as extremely desirable outcomes of youth sport involvement. As research has shown, education *through* the physical (Wood, 1927; Laker, 2001; Gould and Carson, 2008) is an old and well established construct. The idea here being that individuals can learn from their involvement as well as learning the skills necessary to enable it, the latter being education *of* the physical. In both cases, the importance of skills seems explicit and the practical implications or applications completely central to the process. Accordingly, the development and explicit promotion of life skills as a central part of physical education and sport participation would seem an obvious goal. As an important addition, however, one might consider the extent to which these skills also have application to sporting performance itself. in other words, education *for* sport. Crucially, the ideas espoused by our PCDE approach (MacNamara et al., 2010a,b) have already been shown to be effective in driving progress in *both* performance and participation within the *same* developmental environment (Collins et al., 2010).

In summary, we would suggest that there are a number of parallel research strands which offer complimentary support to the skills-based approach. Certainly, contributions to these strands would seem a sensible “value-added” consequence of the skills-based approach.

## Where next? making the super-champ

### What do we need to know?

Given our focus on challenge, its arguably unavoidable presence in TDEs and its nature as a common factor in many of the TD approaches we have mentioned, it would seem sensible to assess how much (volume, intensity, etc.), of what type, and when challenge is optimum to develop a performer's skills and confidence. Clearly, we know that too much anxiety can cause all sorts of problems, with high chronic levels indicated as a precursor to increased risk of cancer for example (Russ et al., 2012; Russ, 2014). Certainly, early life adversity is often not a positive influence for young people (Walsh et al., 2014), which in itself seems to question the genericity of growth through trauma approaches. In short, should we be genuinely concerned at causing undue anxiety in children, even if intentions are positive? Authors such as Stirling and Kerr (2015) have highlighted concerns that stress may generate severe issues for younger athletes, with such problems sometimes emerging well after the initial impositions. Furthermore, they highlight that these challenges may appear completely innocuous at the time. It seems to us that this risk needs careful examination and if appropriate, regulation so that youth sport can generate the sorts of positive outcomes for which it is espoused.

For example, the pressures inherent in Relative Age Effect (RAE) are well documented (e.g., Till et al., 2010; Andronikos et al., 2016). Interestingly, however, there is increasing evidence for a reversal of the RAE benefits, with younger athletes managing to achieve professional status more often than their older peers, even when numerically outnumbered and (at least initially) physically disadvantaged. Investigations highlighting this phenomenon have stressed the role of psychological skills development as the probable mechanism through which these effects occur (McCarthy and Collins, 2014; McCarthy et al., 2016). In simple terms, the extra pressure experienced by the late birth players can generate greater growth, *so long as* appropriate and concurrent support develops the necessary skills. As we have highlighted elsewhere, however, (Collins et al., 2016a) this suggests the need for careful monitoring and thought in applications of pressure with developing athletes, however “transformative” the stated agenda is presented (cf. Bell et al., 2013).

Of course, such careful examination is necessitated for all such processes in youth sport. For the moment, we would suggest that the proactive coping approach integral to the skills development line of PCDEs, coupled with careful consideration (e.g., Hill et al., 2016) and monitoring (e.g., MacNamara and Collins, 2011; Hill et al., in review) of mental health *and* performance issues offers a useful protection against such consequences. Certainly, consideration of the genesis of long term mental health issues in sport (cf. Roberts et al., 2016; Lebrun and Collins, 2017), together with epidemiological work with former academy athletes, are other important areas which we are currently addressing.

For the moment, there are lines of research which offer reassurance. For example, the ongoing work of Savage et al. (e.g., Savage et al., 2016) suggests that the small but significant scale traumas which serve to develop skills in high level performers have a diminishing impact as they learn to cope and develop confidence in their capacity to do so. In short, we have evidence that those who *do* make it up the slippery slope tend to emerge stronger and unblemished. Of course, this requires longitudinal confirmation given that there is a well-documented difference between self-reported growth and actual growth (Cho and Park, 2013). Longitudinal studies are currently underway that distinguish between reported growth (the individual's subjective experience) and actual growth (objective changes) following challenge. Of equal or perhaps even more importance, however, is the careful examination of those who don't emerge stronger. This is an important consideration given that the eventual status of the athlete, whether they succeeded or not, will influence their perception of trauma and challenge.

Another positive line examines the role which parents can play in moderating the stressful impacts of youth sport participation (Tamminen et al., 2016). Of particular relevance, this work emphasized the influence which parental encouragement can have on the employment of active coping strategies. Providing a pleasing counter to the often negative view of parental involvement (e.g., Ross et al., 2015), Tamminen et al.'s work resonates with the PCDE call for “seeking and using social support” as a key skill. Once again, proactive use of the skills in combination can serve to limit negatives and accentuate positives along the developmental pathway.

Of course, and as stated earlier, we also need to employ more longitudinal examinations of TD, tracking developing athletes through their pathway against the development of PCDEs, individuals' experiences of the teach-test cycle and ultimately whether these factors are associated with, or even play a part in, their ultimate success, or lack thereof.

### What sort of research do we need?

Reflecting ideas considered in previous sections, what sort of research is needed to push this important area forwards and optimally realize the clear benefits? Firstly, we would like to see a “pracademic” focus, bridging academic, and practitioner concerns to generate an effective and applicable answer. The term (cf. Battaglio and Scicchitano, 2013) has been in use for a while but we have adopted it to emphasize the pragmatic focus of our work. Such an approach would require researchers to focus on the applied implications of their work, the “so therefore,” which should surely be the primary issue for any agency tasked with enhancing performance. This approach should be coupled with a range of studies, using several methodologies and using prospective and longitudinal designs. We completely acknowledge this weakness in some of our own work and this is one reason why we have built and continue to develop a portfolio of work around the PCDE concept, **a skills-based approach to TD**.

KEY CONCEPT 3 **Evidence driven policy in TD**

We discuss the need for a more open and evidence-based approach to policy and suggest that policy in sport is too often driven by political “neatness” (what makes for a glossy intervention—see Collins et al., 2014) or by extremely secondary sources such as popular books (e.g., Syed, 2010) or social media (MacNamara and Collins, 2015).

KEY CONCEPT 4 **A skills-based approach to TD**

Developing skills in an appropriately challenging environment appears to be a big element of the processes which can lead an athlete to Super-champ status. The main aims of the skills development approach are to build the skill set, develop the performer's ability to deploy subsets of skills to address challenges, and to ensure a confidence and commitment to doing this under pressure.

So, as a simple checklist, research should be project/multi-study rather than single study driven, reflecting a sequence of work which can consider the issue from a variety of angles. Breadth and depth of samples would support this, with a variety of tools used (such as the validation of athlete interviews by parallel coach and parent interviews, cf. Hardy et al., 2013), triangulation wherever possible, longitudinal tracking, and crucially, examination of successes *and* failures. In this regard, the methodological recommendations of Gould and Carson (2008) seem to offer an excellent starting point. Although focused more on the life skills perspective they highlight the need for “(a) quantitative and qualitative research; (b) the development of valid life skills through sport measures; (c) an examination of sport program type differences; (d) evaluation research; (e) longitudinal studies; (f) studies focusing on identifying theoretical explanations for the life skill development sport participation link; (g) the utilization of experimental designs; and (h) an examination of the transferability of life skills” (p. 58).

Lessons may also be drawn from Nutley et al. (2002), whose research offers a cross disciplinary perspective on work aimed at the application of evidence based practice to policy. Their summary offers four guiding principles:

“agreement on what counts as evidence in what circumstances; a strategic approach to the creation of evidence in priority areas, with systematic efforts to accumulate robust bodies of knowledge; effective dissemination of evidence to where it is most needed, and the development of effective means of providing wide access to knowledge; and initiatives to ensure the integration of evidence into policy and encourage the utilization of evidence in practice” (p. 1).

Nutley et al.'s work is focused on public policy in the UK. Unfortunately, their assessment of how well this goal has been met is somewhat pessimistic. As such, it would seem to us that some firm leadership and decision making is needed to ensure that optimum answers are generated and **evidence driven policy in TD** is realised.

As one very simple step, it would seem that truly academic work should consider and address the views of others (cf. Davies et al., 2000). Opponents referring to, and often countering, others research would seem both useful and central to a reasoned argument. We have tried to do this, at least in a small way, within the current paper. In contrast, we find it interesting that others seem to be ignorant (whether blissfully, deliberately, or politely) of our work in their papers. We find this sort of approach to be lacking in academic rigor, especially since we are careful to name and consider (albeit critically) their work. We hope that this paper will stimulate a more critical debate, with authors naming names and addressing contrasts. Without it, progress will surely be limited (Branham, 1991; Tucker, 1995).

## In conclusion—where do we go from here?

In this paper, we have tried to offer a broad ranging coverage of an important area, offering suggestions for progress through honest and evidence-based criticism. We hold no monopoly on veracity but would hope that our position, carefully considered, comprehensively researched across multiple studies and methods, and with demonstrably effective and well received application, can at least be considered as an alternative. Indeed, perhaps we *are* all saying similar things, as per our earlier section. In any case, as we write this, the evidence for and usage of skills based approaches seems to be growing. Supporting development, helping with a wide variety of issues (e.g., injury recovery—Tranaeus et al., 2014a,b) and offering a broad educational benefit for performers—what's not to like?

## Author contributions

All authors listed, have made substantial, direct and intellectual contribution to the work, and approved it for publication.

### Conflict of interest statement

The authors declare that the research was conducted in the absence of any commercial or financial relationships that could be construed as a potential conflict of interest.
